# Advances in Pharmaceutical Science in Electrochemotherapy: A Tribute to Prof. Jolanta Saczko

**DOI:** 10.3390/ph17121718

**Published:** 2024-12-19

**Authors:** Nina Rembiałkowska, Julita Kulbacka

**Affiliations:** 1 Department of Molecular and Cellular Biology, Faculty of Pharmacy, Wroclaw Medical University, Borowska 211A, 50-556 Wroclaw, Poland; julita.kulbacka@umw.edu.pl; 2 State Research Institute Centre for Innovative Medicine, Department of Immunology and Bioelectrochemistry, Santariškių 5, 08410 Vilnius, Lithuania

This Special Issue is dedicated to the memory of Professor Jolanta Saczko (1964–2023), a remarkable leader whose guidance and dedication were instrumental in advancing electroporation-based research in Poland. As the head of our pioneering team, Prof. J. Saczko was vital in fostering groundbreaking studies that laid the foundation for introducing and applying electrochemotherapy in the country. Her efforts in supporting innovative research initiatives and building collaborative networks have significantly shaped the landscape of this therapeutic field, enabling the implementation of electrochemotherapy as a clinical practice in Poland.

Prof. J. Saczko, who served as a Guest Editor for this Special Issue until her passing in 2023, was a visionary leader and mentor. As the Head of the Department of Molecular and Cell Biology at the Faculty of Pharmacy, Wroclaw Medical University, she inspired countless students and colleagues with her scientific rigor, dedication, and compassion. Her medical biochemistry and molecular biology work advanced our understanding of cellular responses to therapies and paved the way for novel treatment modalities.

In this Special Issue of *Pharmaceuticals*, we present five original articles that explore the most recent advancements in pharmaceutical application within the framework of electrochemotherapy. The studies range from in vitro experiments to in vivo investigations, encompassing topics such as gene electrotransfer and the development of innovative therapeutic strategies. This collection is a testament to the continued evolution of pharmaceutical sciences in enhancing the efficacy of electrochemotherapy.

Electrochemotherapy (ECT), which combines electroporation (EP) with standard anticancer agents like bleomycin and cisplatin [1,2,3], is a promising method for triggering additional drug transport pathways into cells. The well-known ECT protocol, following European Standard Operating Procedures (ESOPE), involves applying a burst of 8 × 100 µs, 1 Hz utilizing pulsed electric field (PEF) pulses for targeted drug delivery in cancer removal [4,5,6,7,8,9]. ECT has shown efficacy in treating superficial and deep-seated tumors, including melanoma, breast, liver, and pancreatic tumors [10,11,12,13,14,15].

Electroporation, also called electropermeabilization, is a biophysical technique that transiently increases the permeability of cell membranes under the influence of intense electrical pulses [4,16]. This method has been extensively applied to various cell types, including human, animal, and plant cells, both in vitro and in vivo [17,18,19,20,21] [Contribution 1, Contribution 2]. Creating transient nanoscale pores in the lipid bilayer facilitates the uptake of molecules such as drugs, DNA, RNA, and ions [22,23] [Contribution 3]. Electroporation can be reversible or irreversible (IRE) depending on the intensity and duration of the applied electrical pulses [24,25]. 

Reversible electroporation (EP) allows the cell membrane to reseal after treatment, maintaining cell viability [26,27]. Electroporation, the transient permeabilization of cell membranes using electrical pulses, has emerged as a powerful tool in pharmaceutical and medical science and therapeutic innovation. Its application in electrochemotherapy combines targeted drug delivery with localized electric fields, offering enhanced efficacy for treating resistant malignancies while minimizing systemic toxicity. Used in ECT, cytotoxic agents are usually impermeant in the cells. Bleomycin, a hydrophilic drug with high intrinsic cytotoxicity, is one of the most commonly employed chemotherapeutic agents in ECT, followed by cisplatin [28,29,30]. These drugs penetrate cells more effectively after electroporation, leading to selective tumor cell death without significantly damaging surrounding tissues [24,31].

In contrast, IRE leads to colossal cell membrane damage and subsequent cell lysis. However, using IRE makes this method a promising minimally invasive tissue ablation technique [32,33,34,35] and offers more protocol flexibility and less standardization, including nanosecond bursts of pulses [16,36]. 

While the standard ESOPE protocol employs eight microsecond-long pulses and is widely used in electrochemotherapy, it faces some limitations. These include non-uniform effects on tissue, the risk of thermal damage, and restricted selectivity, particularly in heterogeneous tumor environments. Moreover, the fixed parameters of ESOPE are not readily adaptable to optimize therapeutic outcomes for diverse cancer models. In contrast, nanosecond pulses offer greater flexibility in modulating amplitude, minimize thermal effects, target subcellular structures, and reduce damage to healthy tissues, allowing for effective ECT within larger tumor volumes while remaining within the reversible electroporation range [9,37]. Furthermore, the frequency dependence of tissue impedance emphasizes the suitability of higher-frequency pulses for treating heterogeneous structures [38].

The effectiveness of electroporation is influenced by key parameters, including the duration of pulses (milliseconds, microseconds, or nanoseconds), the total number of pulses delivered, the frequency at which they are applied, the intensity of the electric field, and the configuration of the pulse waveform, whether monopolar or bipolar [3,39,40,41,42,43,44,45]. Each factor must be carefully optimized to achieve the desired biological effect while minimizing unintended damage. Adjusting these parameters allows for precise control over the electroporation process, tailoring it to specific applications, whether intracellular drug delivery, gene transfer, or cell fusion. It was noticed that different cancer cell types display varying susceptibilities to pulsed electric fields, necessitating optimization of treatment protocols [46].

Furthermore, overcoming the reduced efficacy of chemotherapeutics and the growing issue of drug resistance in cancer therapy underscores the importance of combination treatments. Some resistance mechanisms can be overcome by incorporating drugs with distinct mechanisms of action. In addition to classic ECT agents like bleomycin and cisplatin, new combinations with metformin, vinorelbine, and Dp44mT are being explored to enhance therapeutic outcomes and target resistant cancer cell populations [47]. However, new solutions that could overcome drug resistance are still being sought. In recent years, calcium ion-assisted electroporation (CaEP) has emerged as a promising alternative to traditional ECT, where conventional cytostatics have been replaced by commonly available calcium chloride (CaCl_2_) solutions [48,49], demonstrating effectiveness in preclinical and clinical studies [Contribution 4, Contribution 5] [50].

The adaptability and efficacy of electroporation make it a critical tool in developing advanced therapeutic strategies, particularly for cancers with limited treatment options. Its combination with emerging agents and optimization for specific tumor profiles offers a pathway to more effective and personalized oncology therapies.
